# Biochemical Interactions through Microscopic Techniques: Structural and Molecular Characterization

**DOI:** 10.3390/polym14142853

**Published:** 2022-07-13

**Authors:** Hassan Nezammahalleh, Faezeh Ghanati, Shima Rezaei, Mohsin Ali Badshah, Joobee Park, Naseem Abbas, Ahsan Ali

**Affiliations:** 1 Faculty of Biological Science, Tarbiat Modares University, Tehran 14115-111, Iran; nezammahalleh@ut.ac.ir (H.N.); info@hamyarapply.com (F.G.); 2 Research and Development Department, Hamyarapply Group, Tehran 14115-111, Iran; 3 Department of Microbiology, Faculty of Biological Science, Ardebil Branch, Islamic Azad University, Ardebil 5615731567, Iran; rezaiishima@gmail.com; 4 Department of Chemical and Biomolecular Engineering, University of California-Irvine, Irvine, CA 92697, USA; badshahm@uci.edu; 5 Plamica Labs, Batten Hall, 125 Western Ave, Allston, MA 02163, USA; pjoobee@gmail.com; 6 Department of Mechanical Engineering, Sejong University, Seoul 05006, Korea; 7 Department of Mechanical Engineering, Gachon University, Seongnam-si 13120, Korea

**Keywords:** biochemical interactions, fluorescence, material and biological sciences, microscopy, structural and molecular characteristics, resolution scale

## Abstract

Many researchers and scientists have contributed significantly to provide structural and molecular characterizations of biochemical interactions using microscopic techniques in the recent decade, as these biochemical interactions play a crucial role in the production of diverse biomaterials and the organization of biological systems. The properties, activities, and functionalities of the biomaterials and biological systems need to be identified and modified for different purposes in both the material and life sciences. The present study aimed to review the advantages and disadvantages of three main branches of microscopy techniques (optical microscopy, electron microscopy, and scanning probe microscopy) developed for the characterization of these interactions. First, we explain the basic concepts of microscopy and then the breadth of their applicability to different fields of research. This work could be useful for future research works on biochemical self-assembly, biochemical aggregation and localization, biological functionalities, cell viability, live-cell imaging, material stability, and membrane permeability, among others. This understanding is of high importance in rapid, inexpensive, and accurate analysis of biochemical interactions.

## 1. Introduction

Biochemical interactions result in biological structures in both intra- and extra-cellular environments. Structural and molecular characterizations of these interactions are crucial for discovering the underlying science behind biochemical assemblies. Moreover, it is essential to identify the physicochemical properties, activities, and functionalities of biomaterials, as well as tailor them for specific purposes. Given this importance, numerous tools and analytical techniques have been developed to characterize the interactions over the last few decades.

Each tool offers a range of analytical capabilities and can be used for the study of biochemical interactions under specific conditions. Microscopic techniques are powerful tools that can be used to comprehensively characterize the biochemical interactions in different situations [1,2,3,4,5]. These techniques were also integrated with other techniques, such as Raman and IR microscopy, for 3D imaging in biological research with nanometer resolution and high sensitivity (to a single-molecule level) [6,7,8,9,10,11]. For example, IR spectroscopy was used in conjunction with imaging using pixelated IR detectors, e.g., a focal plane array detector, where spectra can be collected from each pixel, resulting in a spectral image with an enormous amount of spatially resolved spectral data. 

Optical microscopic techniques allow us to observe biological structures in intact samples, such as living cells [12,13]. A variety of fluorescent labeling plus a plethora of sophisticated optical microscopy techniques were developed for live-cell imaging [14]: for example, the use of total internal reflection microscopy together with the technique of single-molecule fluorescence resonance energy transfer for real-time observation of biochemical dynamics and interactions [15]. The technique of single-molecule fluorescence microscopy was applied to achieve super-resolution and the visualization of cellular structures in the range of 2 nm [16]. The complex and myriad interactions of biochemicals in cellular membranes were studied using super-resolution microscopy to understand the self-assembly and dynamics of plasma membrane components [17]. Considering the strong sensitivity of biochemical interactions to environmental conditions, light-sheet microscopy was introduced for live 3D imaging [18]. The biological processes can now be observed in their native environments through significant advancements in spatial resolution and the development of novel image processing strategies. The recent advances in microscopy enable us to accurately characterize biochemical interactions down to the molecular level.

Electron microscopes are efficient tools for gaining the structural and chemical information of matter at nano- and atomic-scale resolutions (~0.5 Å) [19,20,21]. Scanning electron microscopy (SEM) and transmission electron microscopy (TEM) are the most prominent microscopic techniques used for the investigation and characterization of objects at the nanoscale with higher accuracy. A focused electron beam is used to scan a sample’s surface with SEM to obtain photographs of the sample [22], whereas TEM is a crucial technique for characterizing one-dimensional (1D) nanostructures because it uses imaging, diffraction, and spectroscopic analysis to reveal details about their shape, crystal structure, and chemical make-up [23]. The recent progress in these tools allows for the study of chemical reactions in liquid media [24]. Furthermore, these tools can complement the study of crystal structures conducted using X-ray crystallography [25].

Scanning probe microscopes provide us with structural and functional insights into biomaterial and cell surfaces [26]. In addition to visualizing morphological features at the molecular level and nanoscale, this analytical tool can be used for the direct measurement, manipulation, and sensing of matter in its native environment [27]. Compared with analytical tools with similar capabilities, such as surface plasmon resonance (SPR), the measurements based on atomic force microscopy are not limited to metallic substrates or sensitive to molecular size [28]. Different analytical techniques for the characterization of biochemical interactions are summarized in Table 1.

Optical microscopy, electron microscopy, and scanning probe microscopy have made significant progress in recent years. A couple of review articles discussed the principles and applications of each one of these microscopic techniques. However, it is essential to figure out the pros and cons of these three main branches of microscopy as a function of the resolution scale (from 250 µm down to 0.05 nm) and the breadth of their applicability. To this end, the microscopy-based characterization of biochemical interactions was studied in the context of material and life sciences in the present work. The findings of this work can be useful to find out the capability of each tool to reach the best solution at the earliest possible time in future works.

## 2. General and Basics Concepts in Microscopy

The light incidence with matter results in the occurrence of five phenomena: reflection, transmission, refraction, diffraction, and the absorption and consequent excitation of fluorescence. The elastically scattered light helps us to distinguish the presence of matter. The latter phenomenon is the absorption of light and consequent emission with a longer wavelength, which can also guide us to the presence of matter by the naked or aided eye.

**Figure 1:**
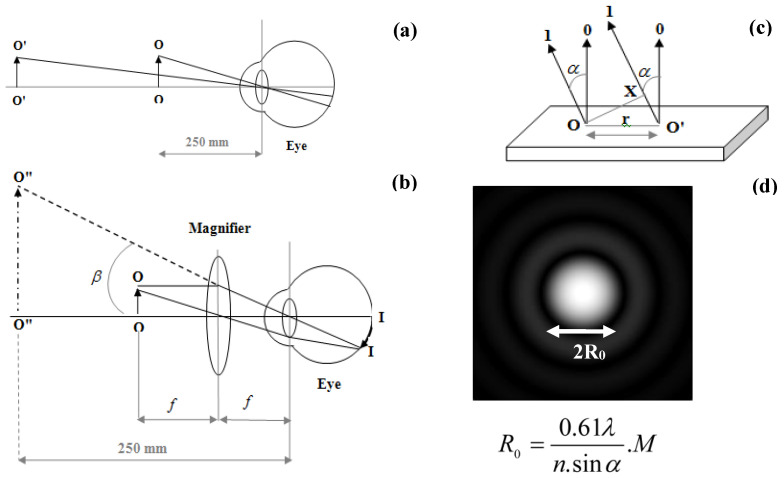
(**a**) Image formation at the eye from a distant object O’-O’ and the one O-O at the nearest distance of distinct vision; (**b**) ray diagram showing the principle of a simple magnifier for increasing the viewing angle of the object O-O and the image of the object on the retina (I-I) formed from the virtual image O”-O” at the nearest distance; (**c**) ray diagram of the zero-order direct light (0) and the 1st-order reflected light (1) from two points separated by r; (**d**) the relationship between the radius of the airy spot and the magnification (M ) by a lens for a point light source.

The basic concepts in microscopy could well be understood by illustrating the image formation in an early optical microscope. Consider an object O’-O’ (Figure 1a) placed at a distance from us, where the light interacts with it and scatters out. The scattered light makes an image on the eye as it falls on the photoreceptor cells in the retina. Two points of the object can be distinguished as distinct if the light from them is separated by at least one cell. The angle formed by the points in the eye is ~1 arcmin. Two points can be resolved only if the viewing angle is increased. To this end, the object must move closer (O-O in Figure 1a) if it is not already at the nearest distance of distinct vision, conventionally taken as 250 mm, or a magnifier should be used (Figure 1b). The object is placed at the focus of a convex lens with a focal length of f. The viewing angle increases to β with a larger retinal image (I-I).

To make a microscopic object visible, the magnification alone would not be sufficient, but instead, it is crucial to resolve fine details and create sufficient contrast. The resolving power can be improved by a suitable choice of objective and the correct setting of a microscope. Sufficient contrast can be created via illumination of the specimen or suitable preparation of the object. Visualization of the object can be done using reflected or transmitted light. The minimum resolved distance is a characteristic of the imaging system. Herein, this Abbe limit of the resolution was calculated for the examination of an object using reflected light [54]. To resolve two points separated by a distance r, the zero-order and at least the first-order diffracted beam must enter the objective lens (Figure 1c). The angle by which the first-order beam is diffracted is indicated by α and the path difference between the zero-order direct light and the first-order diffracted light is the distance O’X, which is equal to r.sinα. This first-order diffracted light must have a constructive interference with direct light. This path difference that gives the first-order constructive interference is equal to 1.λ. Thus, the minimum resolved distance is $r=\frac{\lambda }{1.\sin\alpha }$.

The condenser in a microscope illuminates the specimen in the form of a cone of light with a half angle of α and the space between the lens and the specimen can be any medium with a refractive index n. Under these conditions, the path difference would be 2.r.n.sinα and the limit of resolution is $r=\frac{\lambda }{2.n.\sin\alpha }$. Accordingly, the light source with longer wavelengths is diffracted more, leading to a lower resolution. An objective with a higher numerical aperture (with maximum angle alpha of 70–75°) accepts the higher-order diffracted light, leading to improved resolution.

The maximum spatial resolution of a diffraction-limited optical microscope is ~200 nm in the plane and about 400 nm along the optical axis [55]. The Abbe limit of resolution for the examination of an object using transmitted light is like the one using reflected light.

The specific components in an object can be detected using fluorescent microscopes. In these instruments, the components absorb radiated light, become excited, and emit radiation with lower energy. In contemporary fluorescence microscopes, epifluorescence, which is the fluorescence emitted light, is separated from unabsorbed reflected light, which makes the detection of specific components in matter possible that would not be distinguished by an optical microscope due to the low resolving power. To improve the resolving power of a microscope, electron microscopes were introduced with a much lower wavelength from the illuminating source. However, four categories of optical microscopy methods, including confocal microscopy, interference, non-linear, and surface methods, were developed in the past three decades and improved the spatial resolution down to ~2 nm.

## 3. Biochemical Interactions in Material Sciences

### 3.1. Physicochemical Stability of Nanostructures

**Figure 2:**
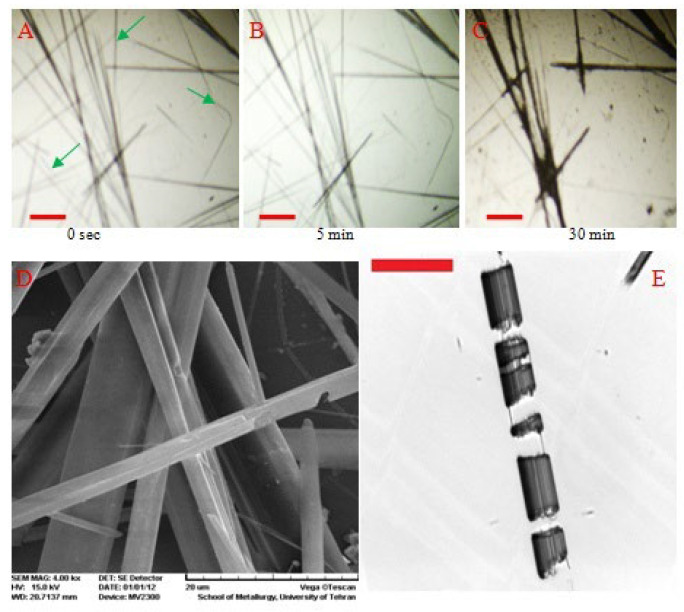
(**A**–**C**) Optical microscopy images of diphenylalanine (FF) micromaterials in the phosphate buffer solution at regular time intervals. Reprinted with permission from [56]. SEM images of the micromaterials after incubation in the buffer solution (**D**) and in methanol (**E**) for a few minutes (reprinted with permission from [58]). Copyright (2015) American Chemical Society. Green arrows indicate completely dissolved nanostructures. Scale bar corresponds to 200 µm.

The chemical stability of nanostructures in a solution can be characterized using an optical microscopy technique with a low resolution of ~250 µm. The constituents of a microstructure interacting with the solvent molecules can begin to dissolve if the free energy of the solvated biochemicals is less than those of their respective components in their pure state. The chemical stability of self-assembled biochemicals in phosphate buffer [56] and many other solvents [57,58] were scrutinized using an optical microscope. To this end, the structural changes of the microstructures were directly monitored under an optical microscope. Moreover, a sample can be observed indirectly via snapshot imaging with scanning electron microscopy (SEM). The preservation of the morphological features after chemical treatment indicates chemical stability, while nanostructure disintegration and morphological changes are the reasons for chemical instability [59]. These changes at the three-dimensional microscale can be well understood using these microscopy techniques, even at low resolution. For example, the dissolution of diphenylalanine (FF) micro/nanostructures in phosphate buffer solution and methanol are discussed below (Figure 2). The optical images were taken at fixed time intervals during the dissolution of the structures in a phosphate buffer (Figure 2A–C). The SEM images of the nanostructures were also taken in the buffer (Figure 2D) and methanol (Figure 2E). The dissolution mechanism was confirmed by analyzing the images. Different solvents would dissolve nanostructures in a chain-type reaction or as power-law nucleation with a consequent interface advance. The former reaction caused the crack development and separation of the nanostructures into pieces (Figure 2E), while the latter led to their thinning (Figure 2D).

### 3.2. Self-Assembly of Biochemicals into Nanostructures

**Figure 3:**
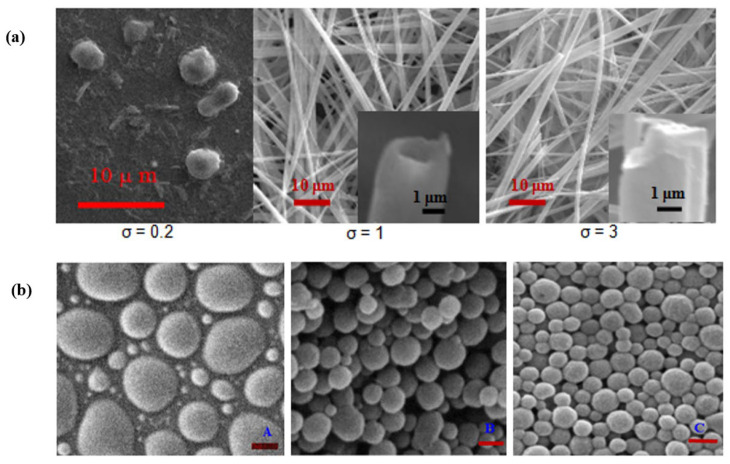
(**a**) Scanning electron micrographs of the diphenylalanine (FF) nanostructures at various degrees of super-saturations; (**b**) SEM images of chitosan-sodium tripolyphosphate/vitamin C nanoparticles at different chitosan molecular weights of 450 (**A**), 110 (**B**), and 65 kDa (**C**), scale bar corresponds to 200 nm; reprinted with permission from [64].

The self-assembly of biochemicals can be characterized using SEM with resolutions <100 µm. For example, the self-assembly of biochemicals, such as FF, in different solvents was monitored to find out the formation of nanostructures for diverse applications [60]. The morphological evolution of the FF into a particular structure is a complex combination of nucleation, growth, habit modification, agglomeration, and aging. These processes are influenced by dominant parameters, such as the degree of super-saturation, the level of active impurities, and the solvent pH. To study the morphological evolution of the FF into a particular nanostructure, an SEM technique was successfully used to find a suitable structure for the correct function (Figure 3a).

For a second example, the self-assembly of biochemicals into nanoparticles was studied using SEM at a resolution of ~100 nm. These types of studies are usually necessary to increase the stability of bioactive nutrients under unfavorable environmental conditions, during the processing and storage of foods, and optimize the release kinetics of the drugs in the body. The shelf life of bioactive nutrients, such as vitamin C [61] and crocin [62], and the delivery of drugs, such as 5-fluorouracil [63], were increased through encapsulation in biodegradable biopolymers via self-assembly. The stability and delivery performance depend on the particle size and morphology. The study of such characteristics of the nanoparticles can all be done via three-dimensional imaging of the structures using electron microscopy techniques. For example, the chitosan molecular weight was associated with the size of the self-assembled nanoparticles of chitosan-sodium tripolyphosphate/vitamin C. The higher the molecular weight, the higher the particle size. The maximum loading efficiency was found to occur at 110 kDa (Figure 3b).

To study the self-assembly of many other biochemicals, it is necessary to equip ourselves with microscopes with higher resolutions of ~10 nm. For example, through the self-assembly of triphenylalanine peptide [65]; diphenylglycine [66]; methyl meta-, ortho-, and para-aminobenzoate [67]; *N*-fluorenylmethoxycarbonyl-diphenylalanine (Fmoc-FF) [68]; and viral capsid proteins with nucleic acids [69] in different media into nanorods, nanospheres, vesicles, and nanofibers, virus-based platforms were found to have possible applications in electronics, tissue engineering, sensing, composites, and drug delivery systems, as shown using transmission electron microscopy (TEM) techniques. Furthermore, TEM was successfully applied to confirm the self-assembly of Fmoc-FF and arginine-glycine-aspartate into nanofibrous hydrogel as a 3D scaffold for anchorage-dependent cells [70].

**Figure 4:**
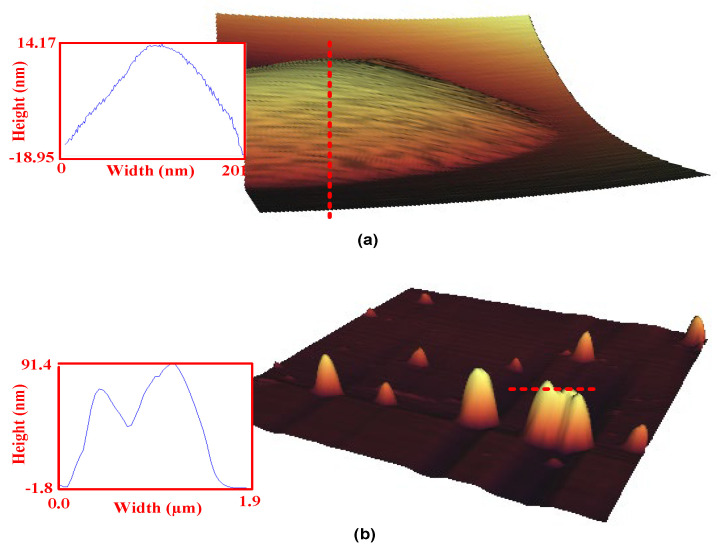
The AFM images of the dipicolinic-acid-imprinted polymers (**a**) and the non-imprinted ones (**b**). The surface topography was elucidated using curves of the density profiles along the red dotted lines. Reprinted with permission from [71].

To reach a much higher resolution of ~1 nm, atomic force microscopy (AFM) was suggested for the study of surface properties. Nezammahalleh et al. [71] used the AFM technique to characterize the surface properties of dipicolinic-acid-imprinted and non-imprinted polymers. The imprinted polymers were found to have surface cavities, while the non-imprinted ones had no significant surface sites for specific binding (Figure 4).

## 4. Biochemical Interactions in Life Sciences

### 4.1. Cyto-Histochemical Studies

**Figure 5:**
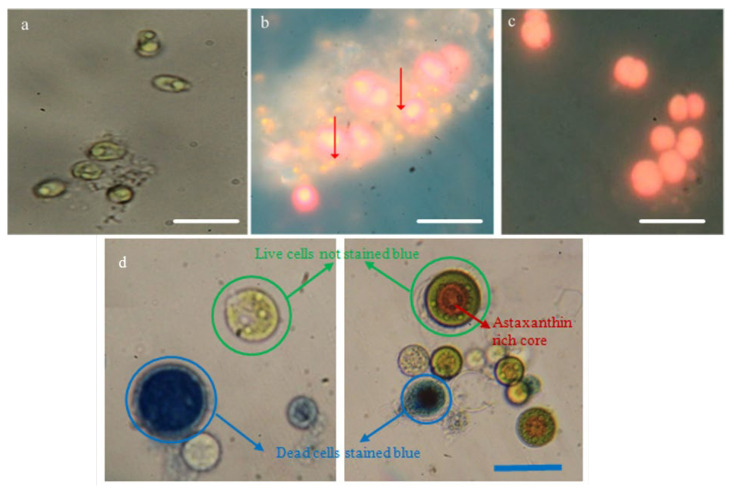
(**a**) Intact *Chlorella vulgaris* cells before staining under an optical microscope. (**b**) Histochemical localization of lipid bodies in the cells after staining with Nile red under a fluorescent microscope. (**c**) The unstained algae under a fluorescent microscope with the characteristic red fluorescence. Red arrows indicate the stained lipid globules emitting fluorescence at 575 nm. Reprinted with permission from [76]. (**d**) Light microscopy images of live versus dead microalgae *Haematococcus pluvialis.* Scale bar corresponds to 20 µm.

The characterization of biochemical interactions is of paramount importance in biological sciences, especially for live-cell imaging. An optical microscope with a low resolution was effectively used for the localization and interaction of biochemicals in a cell using fluorescent biomarkers. To mention a few examples, diphenyl-1-pyrenylphosphine was proposed as the labeling dye for the visualization of lipid and protein hydroperoxides in cells [72]. The cellular lipids can be visualized upon interaction with Nile red dye and the consequent fluorescent emission with variable intensities depends on the surrounding medium (Figure 5). Aqueous media completely quenches fluorescence, while hydrophobic media, e.g., acetone and dimethyl sulfoxide, enhances the fluorescence intensity. The Nile red fluorescence emission can be observed under interaction with plasma membrane lipids and triglyceride lipid globules at 630 nm and 575 nm, respectively [73]. The lipid droplets with yellow fluorescence near the cell wall helped us find the accumulation of lipid globules next to the cell membrane (Figure 5b). The lipid droplets could also be clearly observed in intercellular substances surrounding the cells. This knowledge is very important for in situ lipid extraction from algal species. Unstained *C. vulgaris* cells under a fluorescent microscope show the characteristic red fluorescence from chlorophyll molecules (Figure 5c). The histochemical localization of flavonoids and chlorophylls were successfully observed in microalgal species using an optical microscope with 40X magnification. The flavonoids were known to exist in plants and green algal species [74]. The histochemical examinations of three algal species, including *C. vulgaris*, *Spirulina* sp., and *Fischerella* sp., were carried out using Neu’s reagent. The flavonoid staining with this reagent resulted in bright orange fluorescence under UV light [75]. Histological studies are important in the treatment of living cell species. Table 2 presents a review of different reagents used for the localization of cellular components.

In addition, the optical microscope was effectively used for cell viability assays. One of the main causes of cell death is the irreversible permeability of cellular membranes. The cellular plasma membrane is an active interface for accurate control of metabolic activities and interaction of the cells with the environment. The membrane permeability can be investigated using Evans Blue dye, where dead cells are stained blue after interaction with the dye. This technique was used to determine the number of cells with a disrupted membrane after some physicochemical pretreatment (Figure 5d).

Even though these characterizations are very useful for engineering purposes, they are not applicable to scientific analysis. For example, the study of protein–protein interactions needs much higher resolutions to understand the binding affinity and the kinetics of binding measurements.

### 4.2. Design of Probes for Live Cell Imaging

Over the past few decades, there were significant advances in image processing, as well as in optical instruments that helped us use fluorescent biomarkers for the direct characterization of biochemical interactions down to the molecular level. Compared with non-targeted visualization techniques for engineering purposes (Section 4.1), the target visualization was effective in the characterization of biochemical interactions at much higher resolutions (<50 nm). Lukinavičius et al. [85] designed fluorogenic probes for live-cell imaging of a cytoskeleton in which they develop far-red fluorogenic probes for actin and tubulin fluorescence imaging in living cells that have low cytotoxicity and high brightness and photostability. With a resolution unheard of for visualizing cytoskeletal structures in living cells, they were used in stimulated emission depletion (STED) microscopy to show the ninefold symmetry of the centrosome and the spatial organization of actin in the axon of cultured rat neurons. Similarly, Xu et al. [86] designed a series of aggregation-induced emission-based probes with different electron-donating and electron-withdrawing substituents for hypochlorite detection in real water samples and live-cell imaging. Therefore, designing probes for live-cell imaging plays a crucial role in the detection of cells with a possible lowest limit of detection in multiple diseases.

As a first example, super-resolution microscopy techniques were used to characterize antibody–antigen interactions in cells and whole organisms. This target visualization of different biochemical components is very effective and useful, as there is no interference with protein functions or the cell behavior in response to external stimuli. Recently, nanobodies were also introduced as versatile toolkits for microscopic imaging [87]. The nanobodies were composed of an epitope tag coupled with a fluorescent label using different cross-linking agents. The N-hydroxysuccinimide ester labeling derivates are widely available for designing diverse fluorescent probes. A decrease in the size of the fluorescent biomarkers reduced the distance between the antigen and the fluorescent label and resulted in a much higher resolution. This technique also obviated the need for the over-expression of the proteins and the possibility of interference with normal cell behavior. However, there are several drawbacks to the effective use of these techniques for the characterization of biochemical interactions in live cells. One of the main difficulties is the traversal of nanobodies through the cell membrane without adversely affecting the nanobody functionality or cell behavior.

**Figure 6:**
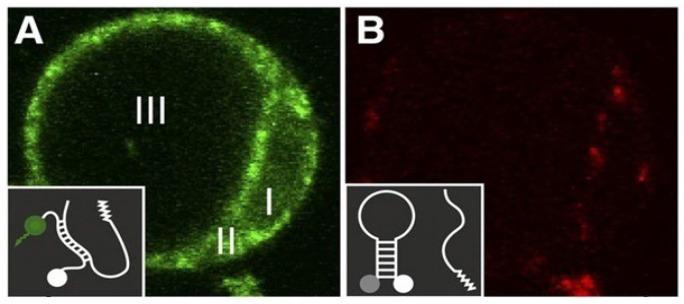
In vivo RNA imaging of Arabidopsis protoplast. Sequence-specific molecular beacons (**A**) and control ones (**B**) were electroporated into the protoplast; I, nuclear region; II, cytoplasm; III, vacuole. Reprinted with permission from [88].

As a second example, we can mention the design of sequence-specific RNA probes for imaging the target RNAs in both living cells and extracellular environments. This characterization is very important for our understanding of cellular RNA metabolism and its functionality. The techniques based on fluorescently labeled RNAs are valuable tools for plant research [88], as well as for many other instances of cell and developmental biology research [89]. For example, molecular beacons (MBs), which are short hairpin-structured nucleic acids with a fluorophore molecule in one end and a quencher one in the other, take two different forms in solutions. In the presence of target RNA, these biochemicals bind with each other and the hairpin stem separates them from the quencher. This hybridization technique was applied for the visualization of the target RNAs under a fluorescence microscope. In a solution without target RNAs, the molecular beacon’s fluorophore group is in proximity to the quencher molecule. This unbounded form of the probe has no fluorescence. Figure 6 shows the introduction of a gene-specific molecular beacon and a control one with no cellular target in an Arabidopsis protoplast. The green fluorescent image of the protoplast was because of the specific interaction of MBs with target RNAs. In this state, the quencher and fluorophore groups were not in proximity, as schematically shown in the inset of Figure 6A. No fluorescence could be observed in the control protoplast (Figure 6B), which was because the MB stem loop brought the fluorophore and quencher groups into proximity, as schematically shown in the inset of Figure 6B.

## 5. Biochemical Interactions at the Interface between Material and Life Sciences

### 5.1. Cell–Nanoparticle Interactions

The electron microscopy techniques can provide us with high-resolution images of cell–material interactions. These techniques do not lead to real-time characterization, but still provide useful information for many scientific and engineering investigations.

The cell–nanoparticle interactions can be visualized using electron microscopy techniques to understand the effects of material charge and size, ultrastructural alterations of cells, and so on. To mention a few examples, Rothen et al. [90] studied the penetration and localization of three different particle types (gold, TiO_2_, and polystyrene materials uncharged or charged negatively and positively) into red blood cells. They concluded that particle size is the most influential factor in the translocation of the particles into the cells. The TEM images of the translocated particles were visualized as black dotted spots. In this article, the LSM micrographs of the particles attached to or within cells were viewed as different colors from the background autofluorescence of the cell. To design efficient drug carriers, the surfaces of silica nanoparticles were modified with different synthetic cell-penetrating peptides and the uptake efficiency by HeLa cells was analyzed using SEM [91].

**Figure 7:**
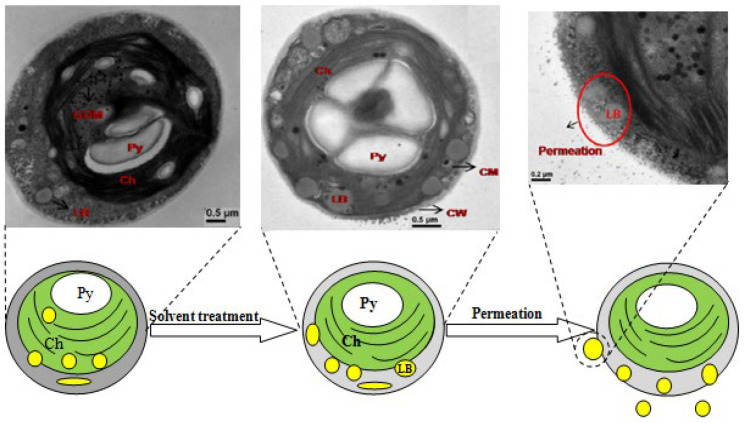
Schematic representation of lipid milking process from *Botryococcus braunii* during treatment with a biocompatible solvent, e.g., tetradecane (Ch: chloroplast; Py: pyrenoid; LB: lipid body; CW: cell wall; CM: cell membrane; GOM: granular osmiophilic material). The upper panel shows the TEM image of the microalgae. Adapted with permission from [96].

In another class of studies, the ultrastructural alterations of the treated cells were characterized using TEM to find out the threshold limit of each treatment and the toxicity mechanism. The uptake of metallic nanoparticles or solvent treatment of microorganisms disrupts their normal cellular functions and changes their ultra-structures [92]. The presence of metallic nanoparticles beyond the threshold limit can disrupt the plasma membrane [93]; disorder grana lamella in photosynthetic cells [93]; and cause cytosol leakage [94], mitochondrial damage [95], and structural organelle damage [92]. All these effects cause cell death with or without inflammation or cell disruption. In situ extraction capability of algal lipids by organic solvents was studied using TEM [96] to understand the structural variation of the cells in the absence or presence of organic solvents (Figure 7). The objective of this study was to find out the possibility of lipid milking. The cell wall structure of algae under lipid milking would be much looser than the control cells and the border of cell walls would also be blurred; meanwhile, structural damage, plasma membrane rupture, and cytosol leakage would occur in algal cells incapable of lipid milking [97].

**Figure 8:**
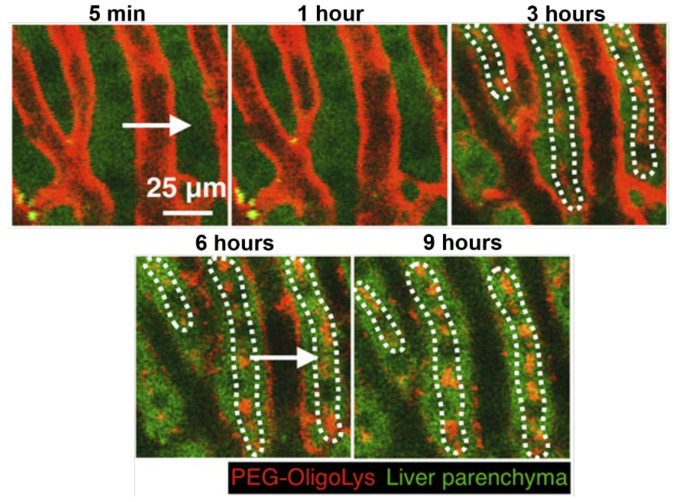
Schematic representation of the interaction of poly(ethylene glycol)-two-arm-PEG-oligo (L-lysine) with liver sinusoidal wall and biliary excretion using IVRT-CLSM with real-time imaging at multiple time intervals of 5 min, 1 h, 3 h, 6 h, and 9 h. Adapted with permission from [53].

In addition, real-time imaging is one of the most valuable analytical tools that allow for direct visualization of the journey and fate of biomaterials and nanoparticles within healthy and diseased organs and cells. Intravital real-time CLSM (IVRT-CLSM) is one such real-time imaging tool that can help to understand the nano-bio interactions in living animals. For example, IVRT-CLSM was used to study the pharmacokinetics, interactions with liver sinusoidal scavenger cells, and biliary elimination of biomaterials and nanoparticles [53]. The interaction of poly(ethylene glycol)-oligo(L-lysine) with liver sinusoidal wall and biliary excretion using IVRT-CLSM is shown in Figure 8 and the trans-endothelial transport of nanoparticles using TEM and intravital microscopy was explained by Sindhwani et al. [47].

### 5.2. Measurement of Biochemical Interaction Forces

**Figure 9:**
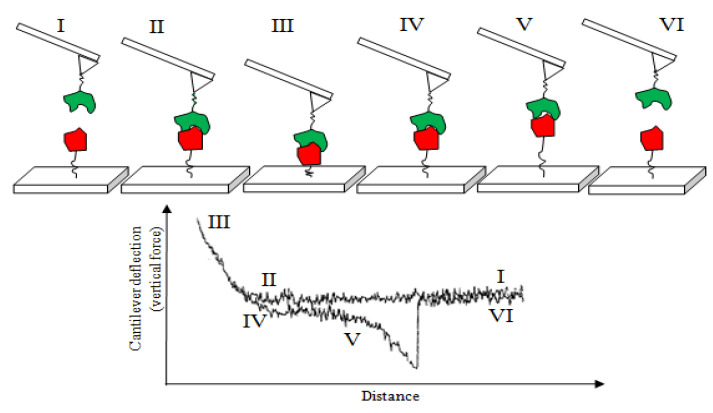
Schematic representation of the interaction between a functionalized AFM tip during attraction toward and retraction away from a chemically modified substrate, along with the corresponding force–distance curves; adapted with permission from [99].

The study of antibody–antigen interaction forces is of high importance for deciphering the mechanisms underlying these biological processes. AFM in force spectroscopy mode is a powerful tool to study such interactions down to the molecular level in an extracellular environment. The microscope tip is functionalized with biochemicals (in single-molecule force spectroscopy) or living cells (in single-cell force spectroscopy). Then, the interactive forces including the adhesion and rupture ones between the functionalized tip and a chemically modified substrate are measured during the attraction and retraction of the tip to/from the surface. The structure and functionality of the biochemicals can be elucidated through this quantitative microscopic technique [98,99], as schematically shown in Figure 9. The states I to III on the curve occur during the tip’s approach toward the substrate. The microscope tip deflects away due to repulsive forces; in state III, the deflection reaches the maximum. The tip is then retracted away from the surface (states IV to VI) and the attractive force decreases continuously. The adhesion bonds progressively loosen until full detachment in state V, where the pulling force of the cantilever exceeds the tip–substrate attractive force. Depending on the number of binding events between the tip and substrate, several characteristic jumps will be observed in the force–distance curve. In this scheme, only one characteristic jump is shown. This technique maps out the distribution of single proteins on a cell surface and provides an understanding of the assembly of surface-associated proteins into functional micro/nano-domains. This characterization technique not only provides us with valuable information on the binding affinity and functionality of proteins but also helps us to discover cell responses under stress conditions [100].

The measurement of biochemical interaction forces using AFM in single-molecule force spectroscopy mode can provide reliable information about the biological activity of sensing systems [101]. The surface of the AFM tip was grafted with the complementary species on the sensing surface. The AFM probes were then used to record the force–distance curves for the two ligand–receptor pairs. The observance of rupture peaks at some tip-to-surface distance can be associated with specific adhesive forces between complementary ligand-receptor molecules [102]. The consistency of the measured forces to the rupture of an antigen–antibody complex was confirmed by comparing the force–distance curves of those recorded between the tip and a clean surface (they were not expected to interact with each other). The main advantage of this technique for verification of the specificity and biological activity of sensing systems is the possibility to carry it out on any substrate with no restriction on size and geometry; in contrast, the study of biological processes using the SPR technique requires a metallic substrate deposited as thin layers on glass [103].

The measurement of interaction forces between different biochemicals using AFM in force spectroscopy mode can provide a wealth of information on cell surface functionalities [104], which benefits drug delivery and cancer research [27,105,106]. These applications of AFM are not discussed further due to brevity.

The AFM imaging technique enables the intact characterization of biochemical interactions at the molecular level. The key benefit of AFM is that we do not need to stain, label, or fix biomaterials or cells on a surface under physiological conditions [100]. However, it is the best technique for visualizing biomaterial surface properties and gaining insights into cell surface functionalities, but it is not applicable to live-cell imaging of intracellular biochemical interactions. The analysis of AFM images was also introduced as a unique tool to acquire valuable information about DNA–protein interactions involved in DNA mismatch repair, as this elucidation process requires purified proteins and no labeling agents [107].

## 6. Conclusions and Future Outlook

Biochemical interactions were found to be of paramount importance in a myriad of scientific research and engineering applications. The morphology of self-assembled biochemicals, stability of nanostructures in different media, determination of binding affinities, kinetics of biochemical interactions, localization of cellular organelles, design of DNA imaging probes, study of cellular structures, and cellular membrane permeability are all crucial research topics for emerging applications in biomedical, food, agricultural, and electronic industries. To this end, microscopic techniques are powerful tools at our disposal.

A broad range of microscopic techniques was developed to conduct biological and physicochemical characterizations. These powerful tools can be applied to confirm the results of other techniques or to discover new facts. The microscopy techniques (such as AFM, TEM, or SEM) can provide direct evidence of morphological features of materials; meanwhile, other techniques (IR, LSR, DLS, and SPR) can provide some indirect evidence of material surface properties. Microscopy is a powerful tool for quantitative and qualitative surface studies in both material and life sciences.

The recent advances in super-resolution or fluorescence microscopy enabled us to run live-cell imaging and improved our understanding of cellular dynamics. However, the high sensitivity of biochemical reactions or cell metabolism to the chemical environments made it impossible to perform accurate observations. It is highly important to find out strategies for live-cell imaging without any external perturbation. In light of image processing algorithms and advanced optical instruments, different fluorescent labels can be applied to visualize several biological processes. However, all these live-cell imaging techniques utilize external compounds, raising concerns about different cellular functions and metabolism than the native intact ones. The external metabolites can induce different cellular pathways. For example, it could lead to peroxide accumulation and different defense mechanism induction, or the over-expression of some biochemicals will change the normal cell behavior. Accordingly, it is necessary to test cellular bioactivity and growth prior to using a specific fluorophore for microscopic analysis. The AFM techniques were successfully applied to live-cell imaging of external cell–material interactions without any need to stain, label, or fix biomaterials or cells on a surface.

The significant advances in super-resolution microscopy allow for mapping out the biochemical organization and self-assembly into different cellular components. However, the application of electron microscopy is strongly suggested for scientific investigations in material science because of the much higher resolution (~0.05 nm). The techniques based on fluorescence microscopy are suggested for the direct observation of cellular dynamics and biochemical functionalities in a different biological milieu. For studies at the interface between the material and life sciences, it is better to rely on AFM techniques. The AFM tips can be modified with different proteins, e.g., antibodies, to characterize biochemical interactions at a high resolution.

## Figures and Tables

**Table 1:** Different analytical techniques for the characterization of biochemical interactions.

Analytical Technique	Applications	Ref.
Circular dichroism spectroscopy (CD)	Determination of protein secondary structures; kinetics and thermodynamics of biochemical interactions; the stability of protein complexes; determination of protein–protein structures	[29,30]
Interferometry	Diagnostic assays; study of protein–protein interactions; kinetic binding measurements;	[31,32]
Dynamic light scattering (DLS)	The size distribution of biomaterials and cells	[33]
Enzyme-linked immunosorbent assay (ELISA)	Detection and quantification of the antigens and antibodies in biological samples	[34]
Infrared and Raman spectroscopy	Molecular dynamics in solution, such as conformation changes during protein–substrate interactions; molecular configurations in cellular environments; biosensing	[11]
Nuclear magnetic resonance spectroscopy (NMR)	Study of weak biochemical interactions, such as protein–protein and protein–ligand interactions; drug design and discovery; identification of structure–activity relationships; identification of key interaction sites of macromolecules	[35,36,37]
Resonance light scattering (RLS)	Study of biochemical assemblies, e.g., porphyrins, in different environments; design of functional supramolecular structures; designation of aggregating species; development of analytical methods	[38,39]
Quartz crystal microbalance (QCM)	Recognition of biochemicals; characterization of enzymatic activities based on biochemical interactions with a chemically modified substrate	[40]
Surface plasmon resonance	Monitoring the affinity-based interaction of biochemicals in different environments; the label-free and real-time detection of pesticides, explosives, bacteria, viruses, toxins, allergens, and biomedical analytes	[41,42,43]
Spectroscopic ellipsometry	Bio-sensing; surface and physical properties of thin-film materials	[44,45]
X-ray crystallography	Study of crystal structures at the level of atomic resolution; identification of the binding modes of biochemical interactions, e.g., protein–ligand interactions; structure-based drug design	[46]
Transmission electron microscopy (TEM)	Structural and chemical characterization, extravasation, and subcellular distribution of particles at the nanoscale with a resolution of 2 nm	[47,48]
Fluorescence correlation spectroscopy (FCS)	FCS can monitor the interactions between biomolecules and nanoparticles, e.g., FCS was used to quantify the functionalization efficiency of ligands (for example, avidin and antibody binding fragments (Fabs)) on the surface of nanoparticles	[49,50]
Confocal laser scanning microscopy (CLSM)	CLSM allows optical slicing through tissues, thus enabling precise real-time imaging of liver cells, organelles, and intracellular trafficking of nanoparticles, such as the endosomal escape ability of nanoparticles	[51]
Intravital real-time CLSM (IVRT-CLSM)	Quantitation of biochemicals, such as H_2_O_2_ and GSH in tissues; extravasation of biomaterials and nanoparticles out of the blood vessels into the tumor area directly in the living animals; IVRT-CLSM is also valuable for understanding the nano-bio interactions, such as sequestration and fate of biomaterials and nanoparticles in the reticuloendothelial system organs, such as biliary excretion	[47,52,53]

**Table 2:** Histological observation of living cells under different environmental conditions.

Biochemical	Reagent	Remarks	Ref.
Adenosine triphosphate (ATP)	D-luciferin/luciferase	The luciferase oxidizes D-luciferin in the presence of ATP and magnesium to enzyme-bound luciferil-adenylate. The luciferil-adenylate complex is subsequently oxidized to oxyluciferine. The light emission is a consequence of the rapid loss of energy of the oxyluciferine molecule from an excited state to a stable one such that yellow-green photons are emitted. The amount of emitted light is proportional to the ATP content.	[77,78]
Callose	Aniline blue	The reagent stains the callose in plant cell walls blue, which can be visualized under a light microscope.	[79]
Lignin	Phloroglucinol + HCl	The reagent stains lignin purple-red, which can be visualized under a light microscope.	[80]
Lipase	Resorufin ester	The resorufin ester has no fluorescence, while its cleavage via lipase enzymatic action releases resorufin, which emits fluorescence under visible light excitation at 570 nm in protoplast.	[81]
Lipid bodies	Nile red	The reagent specifically stains lipid globules red, which can be visualized under a fluorescence microscope.	[76]
Protein reserves	Naphthol Blue Black	The reagent specifically stains protein reserves dark blue, which can be visualized under a light microscope.	[82]
Nucleic acids	Alum hematoxylin	The reagent stains the nuclei blue, which is observable under a light microscope. This staining procedure is followed by counterstaining with an alcoholic solution of eosin Y, which stains other cellular structures red, pink, and orange.	[83]
Starch reserves	Periodic acid–Schiff	The reagent stains starch reserves pink, which can be visualized under a light microscope.	[74]
Suberin	Toluidine blue O	The reagent stains the aliphatic domains of suberin yellow, which can be visualized with an optical microscope using white light.	[84]

## Data Availability

Not applicable.

## References

[1] Zhang Q., Cheng Z., Wang Y., Fu L. (2021). Dietary protein-phenolic interactions: Characterization, biochemical-physiological consequences, and potential food applications. Crit. Rev. Food Sci. Nutr..

[2] Danielsen S.P., Beech H.K., Wang S., El-Zaatari B.M., Wang X., Sapir L., Ouchi T., Wang Z., Johnson P.N., Hu Y. (2021). Molecular characterization of polymer networks. Chem. Rev..

[3] Badshah M.A., Koh N.Y., Zia A.W., Abbas N., Zahra Z., Saleem M.W. (2020). Recent developments in plasmonic nanostructures for metal enhanced fluorescence-based biosensing. Nanomaterials.

[4] Abbas N., Hussain M., Zahra N., Ahmad H., Muhammad S., Mehdi Z., Sajjad U., Amer M. (2020). Optimization of Cr seed layer effect for surface roughness of As-deposited silver film using electron beam deposition method. J. Chem. Soc. Pak..

[5] Abbas N., Shad M.R., Hussain M., Mehdi S.M.Z., Sajjad U. (2019). Fabrication and characterization of silver thin films using physical vapor deposition, and the investigation of annealing effects on their structures. Mater. Res. Express.

[6] Yong Y.-C., Wang Y.-Z., Zhong J.-J. (2018). Nano-spectroscopic imaging of proteins with near-field scanning optical microscopy (NSOM). Curr. Opin. Biotechnol..

[7] Baldock S.J., Talari A.C.S., Smith R., Wright K.L., Ashton L. (2019). Single-cell Raman microscopy of microengineered cell scaffolds. J. Raman Spectrosc..

[8] Ao J., Fang X., Miao X., Ling J., Kang H., Park S., Wu C., Ji M. (2021). Switchable stimulated Raman scattering microscopy with photochromic vibrational probes. Nat. Commun..

[9] Xiong H., Qian N., Miao Y., Zhao Z., Chen C., Min W. (2021). Super-resolution vibrational microscopy by stimulated Raman excited fluorescence. Light Sci. Appl..

[10] Cheng Q., Miao Y., Wild J., Min W., Yang Y. (2021). Emerging applications of stimulated Raman scattering microscopy in materials science. Matter.

[11] Badshah M.A., Kim J., Yeom J., Abbas N., Haq M.R., Kim Y., Lu X., Kim S.-M. (2021). Glass nanoimprinted plasmonic nanostructure for high power laser stable surface-enhanced Raman spectroscopy substrate. Appl. Surf. Sci..

[12] Sarkans U., Chiu W., Collinson L., Darrow M.C., Ellenberg J., Grunwald D., Hériché J.-K., Iudin A., Martins G.G., Meehan T. (2021). REMBI: Recommended Metadata for Biological Images—enabling reuse of microscopy data in biology. Nat. Methods.

[13] Wang Z., Zhu L., Zhang H., Li G., Yi C., Li Y., Yang Y., Ding Y., Zhen M., Gao S. (2021). Real-time volumetric reconstruction of biological dynamics with light-field microscopy and deep learning. Nat. Methods.

[14] Stephens D.J., Allan V.J. (2003). Light microscopy techniques for live cell imaging. Science.

[15] Roy R., Hohng S., Ha T. (2008). A practical guide to single-molecule FRET. Nat. Methods.

[16] Moerner W.E. (2012). Microscopy beyond the diffraction limit using actively controlled single molecules. J. Microsc..

[17] Stone M.B., Shelby S.A., Veatch S.L. (2017). Super-resolution microscopy: Shedding light on the cellular plasma membrane. Chem. Rev..

[18] Hansel C.S., Holme M.N., Gopal S., Stevens M.M. (2020). Advances in high-resolution microscopy for the study of intracellular interactions with biomaterials. Biomaterials.

[19] Kuei B., Aplan M.P., Litofsky J.H., Gomez E.D. (2020). New opportunities in transmission electron microscopy of polymers. Mater. Sci. Eng. R: Rep..

[20] Abbas N., Lu X., Badshah M.A., In J.B., Heo W.I., Park K.Y., Lee M.-K., Kim C.H., Kang P., Chang W.-J. (2018). Development of a protein microarray chip with enhanced fluorescence for identification of semen and vaginal fluid. Sensors.

[21] Badshah M.A., Ju J., Lu X., Abbas N., Kim S.-M. (2018). Enhancing the sensitivity of DNA microarrays by metal-enhanced fluorescence using vertical nanorod structures. Sens. Actuators B Chem..

[22] Laws R., Steel D.H., Rajan N. (2022). Research techniques made simple: Volume scanning electron microscopy. J. Investig. Dermatol..

[23] Egerton R., Watanabe M. (2022). Spatial resolution in transmission electron microscopy. Micron.

[24] Kashin A.S., Ananikov V.P. (2019). Monitoring chemical reactions in liquid media using electron microscopy. Nat. Rev. Chem..

[25] Sunde M., Blake C. (1997). The structure of amyloid fibrils by electron microscopy and X-ray diffraction. Adv. Protein Chem..

[26] Hu Y., Ju B. (2021). Non-equidistant scanning path generation for the evaluation of surface curvature in metrological scanning probe microscopes. Meas. Sci. Technol.

[27] Variola F. (2015). Atomic force microscopy in biomaterials surface science. Phys. Chem. Chem. Phys..

[28] Cecchet F., Duwez A.-S., Gabriel S., Jerome C., Jerome R., Glinel K., Demoustier-Champagne S., Jonas A.M., Nysten B. (2007). Atomic force microscopy investigation of the morphology and the biological activity of protein-modified surfaces bor bio and immunosensors. Anal. Chem..

[29] Miles A.J., Wallace B.A. (2020). Biopharmaceutical Applications of Protein Characterization by Circular Dichroism Spectroscopy. Biophysical Characterization of Proteins in Developing Biopharmaceuticals.

[30] Honisch C., Donadello V., Hussain R., Peterle D., De Filippis V., Arrigoni G., Gatto C., Giurgola L., Siligardi G., Ruzza P. (2020). Application of circular dichroism and fluorescence spectroscopies to assess photostability of water-soluble porcine lens proteins. ACS Omega.

[31] Desai M., Di R., Fan H. (2019). Application of biolayer interferometry (BLI) for studying protein-protein interactions in transcription. J. Vis. Exp..

[32] Abbas S., Koch K.W. (2021). Label-free quantification of direct protein-protein interactions with backscattering interferometry. Bio-Protoc..

[33] Kumar A., Dubey R., Singhai S., Dutt Konar A., Basu A. (2020). Structural characterization with light scattering: A tool for rationally designing protein formulations. Anal. Biochem..

[34] Gan S.D., Patel K.R. (2013). Enzyme immunoassay and enzyme-linked immunosorbent assay. J. Investig. Dermatol..

[35] Pellecchia M. (2005). Solution nuclear magnetic resonance spectroscopy techniques for probing intermolecular interactions. Chem. Biol..

[36] Takeuchi K., Wagner G. (2006). NMR studies of protein interactions. Curr. Opin. Struct. Biol..

[37] Barile E., Pellecchia M. (2014). NMR-based approaches for the identification and optimization of inhibitors of protein-protein interactions. Chem. Rev..

[38] Huang C.Z., Li Y.F. (2003). Resonance light scattering technique used for biochemical and pharmaceutical analysis. Anal. Chem. Acta.

[39] Ju P., Zhang Y., Ding J., Zheng Y., Wang S., Jiang F., Sun C. (2021). New insights into the toxic interactions of polyvinyl chloride microplastics with bovine serum albumin. Environ. Sci. Pollut. Res..

[40] Cheng C.I., Chang Y.-P., Chu Y.-H. (2012). Biomolecular interactions and tools for their recognition: Focus on the quartz crystal microbalance and its diverse surface chemistries and applications. Chem. Soc. Rev..

[41] Kim J., Abbas N., Lee S., Yeom J., Asgar M.A., Badshah M.A., Lu X., Kim Y.K., Kim S.-M. (2020). Fabrication of a Plasmonic Nanoantenna Array Using Metal Deposition on Polymer Nanoimprinted Nanodots for an Enhanced Fluorescence Substrate. Polymers.

[42] Lu X., Lee S., Kim J., Abbas N., Badshah M.A., Kim S.-M. (2021). Fabrication of Ag nanorods on micropost array for a metal-enhanced fluorescence substrate with a high signal-to-background ratio. Biosens. Bioelectron..

[43] Badshah M.A., Michel D., Alam N.E., Madni I., Abbas N., Alameh K., Kim S.-M. (2020). Enhancing the Sensitivity of a Surface Plasmon Resonance Sensor with Glancing Angle Deposited Nanostructures. Plasmonics.

[44] Plikusiene L., Maciulis V., Ramanavicius A., Ramanaviciene A. (2022). Spectroscopic ellipsometry and quartz crystal microbalance with dissipation for the assessment of polymer layers and for the application in biosensing. Polymers.

[45] Tanovska M., Rahmani M., Vladimirova-Mihaleva L., Berger M., Neshev D., Momchilova A., Tzoneva R. (2019). An ellipsometric study of interaction of anti-cancer agent erufosine on lipid model systems. AIP Conf. Proc..

[46] McNae I.W., Kan D., Kontopidis G., Patterson A., Taylor P., Worrall L., Walkinshaw M.D. (2005). Studying protein-ligand interactions using protein crystallography. Crystallogr. Rev..

[47] Sindhwani S., Syed A.M., Ngai J., Kingston B.R., Maiorino L., Rothschild J., MacMillan P., Zhang Y., Rajesh N.U., Hoang T. (2020). The entry of nanoparticles into solid tumours. Nat. Mater..

[48] Perche F., Yi Y., Hespel L., Mi P., Dirisala A., Cabral H., Miyata K., Kataoka K. (2016). Hydroxychloroquine-conjugated gold nanoparticles for improved siRNA activity. Biomaterials.

[49] Engelberg Y., Ragonis-Bachar P., Landau M. (2022). Rare by Natural Selection: Disulfide-Bonded Supramolecular Antimicrobial Peptides. Biomacromolecules.

[50] Schmitt S., Nuhn L., Barz M., Butt H.J., Koynov K. (2022). Shining light on polymeric drug nanocarriers with fluorescence correlation spectroscopy. Macromol. Rapid Commun..

[51] Dirisala A., Uchida S., Li J., Van Guyse J.F., Hayashi K., Vummaleti S.V., Kaur S., Mochida Y., Fukushima S., Kataoka K. (2022). Effective mRNA Protection by Poly (l-ornithine) Synergizes with Endosomal Escape Functionality of a Charge-Conversion Polymer toward Maximizing mRNA Introduction Efficiency. Macromol. Rapid Commun..

[52] Li J., Dirisala A., Ge Z., Wang Y., Yin W., Ke W., Toh K., Xie J., Matsumoto Y., Anraku Y. (2017). Therapeutic Vesicular Nanoreactors with Tumor-Specific Activation and Self-Destruction for Synergistic Tumor Ablation. Angew. Chem..

[53] Dirisala A., Uchida S., Toh K., Li J., Osawa S., Tockary T.A., Liu X., Abbasi S., Hayashi K., Mochida Y. (2020). Transient stealth coating of liver sinusoidal wall by anchoring two-armed PEG for retargeting nanomedicines. Sci. Adv..

[54] Bradbury S., Bracegirdle B. (1998). Introduction to Light Microscopy.

[55] Spector D.L., Goldman R.D. (2006). Basic Methods in Microscopy.

[56] Nezammahalleh H., Amoabediny G., Kashanian F., Foroughi Moghaddam M.H. (2015). An investigation on the chemical stability and a novel strategy for long term stabilization of diphenylalanine nanostructures in aqueous solution. Results Phys..

[57] Andersen K.B., Castillo-Leon J., Hedstrom M., Svendsen E. (2011). Stability of diphenylalanine peptide nanotubes in solution. Nanoscale.

[58] Mason T.O., Chirgadze D.Y., Levin A., Adler-Abramovich L., Gazit E., Knowles T.P.J., Buell A.K. (2014). Expanding the solvent chemical space for self-assembly of dipeptide nanostructures. ACS Nano.

[59] Ryu J., Park C.B. (2010). High stability of self-assembled peptide nanowires against thermal, chemical, and proteolytic attacks. Biotechnol. Bioeng..

[60] Yan X., Zhu P., Li J. (2010). Self-assembly and application of diphenylalanine-based nanostructures. Chem. Soc. Rev..

[61] Alishahi A., Mirvaghefi A., Tehrani M.R., Farahmand H., Shojaosadati S.A., Dorkoosh F.A., Elsabee M.Z. (2011). Shelf life and delivery enhancement of vitamin C using chitosan nanoparticles. Food Chem..

[62] Rahaiee S., Shojaosadati S.A., Hashemi M., Moini S., Razavi S.H. (2015). Improvement of crocin stability by biodegradable nanoparticles of chitosan-alginate. Int. J. Biol. Macromol..

[63] Maghsoudi A., Shojaosadati S.A., Vasheghani Farahani E. (2008). 5-Fluorouracil-loaded BSA nanoparticles: Formulation optimization and in vitro release study. AAPS PharmSciTech.

[64] Nezammahalleh H., Amoabediny G. (2013). Novel method for prediction of micro/nanostructures of diphenylalanine dipeptide based on semiempirical thermodynamic study. Fluid Phase Equilibria.

[65] Guo C., Luo Y., Zhou R., Wei G. (2014). Triphenylalanine peptides self-assemble into nanospheres and nanorods that are different from the nanovesicles and nanotubes formed by diphenylalanine peptides. Nanoscale.

[66] Reches M., Gazit E. (2004). Formation of closed cage nanostructures by self assembly of aromatic dipeptides. Nano Lett..

[67] Koley P., Pramanik A. (2011). Nanostructures from single amino acid-based molecules: Stability, fibrillation, encapsulation, and fabrication of silver nanoparticles. Adv. Funct. Mater..

[68] Kim J.H., Lim S.Y., Nam D.H., Ryu J., Ku S.H., Park C.B. (2011). Self-assembled, photoluminescent peptide hydrogel as a versatile platform for enzyme-based optical biosensors. Biosens. Bioelectron..

[69] Luque D., Caston J.R. (2020). Cryo-electron microscopy for the study of virus assembly. Nat. Chem. Biol..

[70] Zhou M., Smith A.M., Das A.K., Hodson N.W., Collins R.F., Ulijn R.V., Gough J.E. (2009). Self-assembled peptide based hydrogels as scaffolds for anchorage dependent cells. Biomaterials.

[71] Nezammahalleh H., Mousavizadeh S.H., Babaeipour V. (2018). New potentiometric sensor based on molecularly imprinted polymer for dipicolinic acid detection in aqueous media. IEEE Sens..

[72] Cropotova J., Rustad T. (2020). A new fluorimetric method for simultaneous determination of lipid and protein hydroperoxides in muscle foods with the use of diphenyl-1-pyrenylphosphine (DPPP). LWT-Food Sci. Technol..

[73] Pick U., Rachutin-Zalogin T. (2012). Kinetic anomalies in the interactions of Nile red with microalgae. J. Microbiol. Methods.

[74] Markham K.R., Porter L.J. (1969). Flavonoids in the green algae (chlorophyta). Phytochemistry.

[75] Dihazi A., Serghini M.A., Jaiti F., Daayf F., Driouich A., Dihazi H., El Hadrami I. (2011). Structural and biochemical changes in salicylic acid treated date palm roots challenged with Fusarium oxysporum f. sp. albedinis. J. Pathog..

[76] Nezammahalleh H., Nosrati M., Ghanati F., Shojaosadati S.A. (2017). Exergy based screening of biocompatible solvents for in situ lipid extraction from Chlorella vulgaris. J. Appl. Phycol..

[77] Chollet R., Ribault S. (2012). Use of ATP bioluminescence for rapid detection and enumeration of contaminants: The milliflex rapid microbiology detection and enumeration system. Bioluminescence-Recent Advances in Oceanic Measurements and Laboratory Applications.

[78] Chollet R., Kukuczka M., Halter N., Romieux M., Marc F., Meder H., Beguin V., Ribault S. (2008). Rapid detection and enumeration of contaminants by ATP bioluminescence using the Milliflex rapid microbiology detection and enumeration system. J. Rapid Methods Autom. Microbiol..

[79] Gahan P.B. (1984). Plant Hustochemistry and Cytochemistry: An Introduction.

[80] Southerton S.G., Deverall B.J. (1990). Histochemical and chemical evidance for lignin accumulation during the expression of resistance to leaf rust fungi in wheat. Physiol. Mol. Plant Pathol..

[81] Gupta A., Sadeghipour H.R., Bhatla S.C. (2003). Subcellular detection of lipase activity in plant protoplasts using fluorescence microscopy. Plant Growth Regul..

[82] Fisher D.B. (1968). Protein staining of ribboned epon sections for light microscopy. Histochemie.

[83] Llewellyn B.D. (2009). Nuclear staining with alum hematoxylin. Biotech. Histochem..

[84] Ghanati F., Morita A., Yokota H. (2005). Deposition of suberin in roots of soybean induced by excess boron. Plant Sci..

[85] Kumari A., Kesarwani S., Javoor M.G., Vinothkumar K.R., Sirajuddin M. (2020). Structural insights into filament recognition by cellular actin markers. EMBO J..

[86] Xu C., Zhou Y., Li Z., Zhou Y., Liu X., Peng X. (2021). Rational design of AIE-based fluorescent probes for hypochlorite detection in real water samples and live cell imaging. J. Hazard. Mater..

[87] Beghein E., Gettemans J. (2017). Nanobody technology: A versatile toolkit for microscopic imaging, protein-protein interaction analysis, and protein function exploration. Front. Immunol..

[88] Gohring J., Jacak J., Barta A. (2014). Imaging of endogenous messenger RNA splice variants in living cells reveals nuclear retention of transcripts inaccessible to nonsense-mediated decay in Arabidopsis. Plant Cell.

[89] Tilsner J. (2015). Techniques for RNA in vivo imaging in plants. J. Microsc..

[90] Rothen-Rutishauser B.M., Schürch S., Haenni B., Kapp N., Gehr P. (2006). Interaction of fine particles and nanoparticles with red blood cells visualized with advanced microscopic techniques. Environ. Sci. Technol..

[91] Gessner I., Klimpel A., Klubmann M., Neundorf I., Mathur S. (2020). Interdependance of charge and secondary structure on cellular uptake of cell penetrating functionalized silica nanoparticles. Nanoscale Adv..

[92] Attarilar S., Yang J., Ebrahimi M., Wang Q., Liu J., Tang Y., Yang J. (2020). The toxicity phenomenon and the related occurrence in metal and metal oxide nanoparticles: A review from the biomedical perspective. Front. Bioeng. Biotechnol..

[93] Gong N., Shao K., Feng W., Lin Z., Liang C., Sun Y. (2011). Biotoxicity of nickel oxide nanoparticles and bioremediation by microalgae Chlorella vulgaris. Chemosphere.

[94] Li W.-R., Xie X.-B., Shi Q.-S., Zeng H.-Y., OU-Yang Y.-S., Chen Y.-B. (2010). Antibacterial activity and mechanism of silver nanoparticles on Escherichia coli. Appl. Microbiol. Biotechnol..

[95] Jin C., Tang Y., Yang F.G., Li X.L., Xu S., Fan X.Y., Huang Y.Y., Yang Y.J. (2011). Cellular toxicity of TiO2 nanoparticles in anatase and rutile crystal phase. Biol. Trace Elem. Res..

[96] Zhang F., Cheng L.-H., Gao W.-L., Xu X.-H., Zhang L., Chen H.-L. (2011). Mechanism of lipid extraction from botryococcus braunii FACHB 357 in a biphasic bioreactor. J. Biotechnol..

[97] Kleinegris D.M.M., Janssen M., Brandenburg W.A., Wijffels R.H. (2011). Two-phase systems: Potential for in situ extraction of microalgal products. Biotechnol. Adv..

[98] Hu X., Li H. (2014). Force spectroscopy studies on protein-ligand interactions: A single protein mechanics perspective. FEBS Lett..

[99] Willemsen O.H., Snel M.M.E., Cambi A., Greve J., De Grooth B.G., Figdor C.G. (2000). Biomolecular interactions measured by atomic force microscopy. Biophys. J..

[100] Dufrene Y.F. (2014). Atomic force microscopy in microbiology: New structural and functional insights into the microbial cell surface. MBio.

[101] Johnson K.C., Thomas W.E. (2018). How do we know when single-molecule force spectroscopy really tests single bonds?. Biophys. J..

[102] Wang C., Hu R., Morrissey J.J., Kharasch E.D., Singamaneni S. (2017). Single moleculae force spectroscopy to compare natural versus artificial antibody-antigen interaction. Small.

[103] Zhou X.-L., Yang Y., Wang S., Liu X.-W. (2019). Surface plasmon resonance microscopy: From single-molecule sensing to single-cell imaging. Angew. Chem..

[104] Shan Y., Wang H. (2015). The structure and function of cell membranes examined by atomic force microscopy and single-molecule force spectroscopy. Chem. Soc. Rev..

[105] Longo G., Kasas S. (2014). Effects of antibacterial agents and drugs monitored by atomic force microscopy. Wiley Interdiscip. Rev. Nanomed. Nanobiotechnol..

[106] Omidvar R., Tafazzoli-shadpour M., Shokrgozar M.A., Rostami M. (2014). Atomic force microscope-based single cell force spectroscopy of breast cancer cell lines: An approach for evaluating cellular invasion. J. Biomech..

[107] LeBlanc S., Wilkins H., Li Z., Kaur P., Wang H., Erie D.A. (2017). Using atomic force microscopy to characterize the conformational properties of proteins and protein-DNA complexes that carry out DNA repair. Methods Enzymol..

